# Challenges and lessons learned during the planning and early implementation of the RTS,S/AS01_E_ malaria vaccine in three regions of Ghana: a qualitative study

**DOI:** 10.1186/s12936-022-04168-9

**Published:** 2022-05-12

**Authors:** Jane Grant, Thomas Gyan, Francis Agbokey, Jayne Webster, Brian Greenwood, Kwaku Poku Asante

**Affiliations:** 1 Faculty of Infectious and Tropical Diseases, London School of Hygiene and Tropical Medicine, Keppel St., London, WC1E 7HT UK; 2 Research and Development Division, Kintampo Health Research Centre, Kintampo North Municipality, Kintampo, Ghana

**Keywords:** RTS,S, Malaria, Malaria Vaccine Implementation Programme, Vaccine introduction, CFIR, Ghana

## Abstract

**Background:**

In 2019, the RTS,S/AS01_E_ malaria vaccine was introduced on a pilot basis in six regions of Ghana by the Ministry of Health/Ghana Health Service as part of the WHO-coordinated Malaria Vaccine Implementation Programme (MVIP). This is the first time a malaria vaccination programme has been implemented in any country. This paper describes the challenges faced, and lessons learned, during the planning and early implementation of the RTS,S/AS01_E_ vaccine in three out of the six regions that implemented the programme in Ghana.

**Methods:**

Twenty-one in-depth interviews were conducted with regional and district health service managers and frontline health workers three months after the start of MVIP in May 2019. Data were coded using NVivo software version 12 and a coding framework was developed to support thematic analysis to identify the challenges and lessons learned during the RTS,S/AS01_E_ implementation pilot, which were also categorized into the Consolidated Framework for Implementation Research (CFIR).

**Results:**

Participants reported challenges related to the characteristics of the intervention, such as issues with the vaccine schedule and eligibility criteria, and challenges related to how it was implemented as a pilot programme. Additionally, major challenges were faced due to the spread of rumours leading to vaccine refusals; thus, the outer setting of the CFIR was adapted to accommodate rumours within the community context. Health service managers and frontline health workers also experienced challenges with the process of implementing RTS,S/AS01_E_, including inadequate sensitization and training, as well as issues with the timeline. They also experienced challenges associated with the features of the systems within which the vaccine was being implemented, including inadequate resources for cold-chain at the health facility level and transportation at the district and health facility levels. This study identified the need for a longer, more intensive and sustained delivery of contextually-appropriate sensitization prior to implementation of a programme such as MVIP.

**Conclusions:**

This study identified 12 main challenges and lessons learned by health service managers and health workers during the planning and early implementation phases of the RTS,S/AS01_E_ pilot introduction in Ghana. These findings are highly relevant to the likely scale-up of RTS,S/AS01_E_ within Ghana and possible implementation in other African countries, as well as to other future introductions of novel vaccines.

## Background

In 2019, an estimated 229 million malaria cases resulted in 409,000 deaths, the majority of these deaths occurring in young sub-Saharan African children [1]. These deaths occurred despite significant progress in malaria control since the year 2000, with the implementation and scale-up of multiple preventative and curative interventions. In recent years, progress in malaria control has plateaued in several countries, creating an urgent need to develop and implement new strategies [1].

The RTS,S/AS01_E_ is the first malaria vaccine to be deployed widely and has been shown to provide partial protection against uncomplicated and severe malaria in young children in a phase 3 trial [2]. In 2016, the World Health Organization (WHO) recognized the potential public health impact of RTS,S/AS01_E_ and recommended the pilot implementation of the vaccine in three to five sub-Saharan African settings [3]. Following this recommendation, a country-led, WHO-coordinated Malaria Vaccine Implementation Programme (MVIP) was established to support the pilot implementation of the vaccine in routine settings. Ghana, Kenya and Malawi were selected to introduce RTS,S/AS01_E_ through their national immunization programmes, reaching 360,000 children per year [4].

In April 2019, the Ghanaian Ministry of Health/Ghana Health Service launched the MVIP in Ghana, with administration of the vaccine through the Expanded Programme on Immunization (EPI) into six regions starting on May 1st 2019 [5]. Alongside the introduction of the vaccine into the routine immunization system, a team of WHO and in-country and international researchers are evaluating the programme to assess the feasibility, safety and impact of the RTS,S/AS01_E_ introduction. As part of the evaluation, only selected areas within the regions introduced the vaccine, while other districts served as comparison areas. The findings from the MVIP were reviewed in 2021 and WHO formally recommended the use of the RTS,S/AS01_E_ vaccine for children living in regions with moderate to high malaria transmission [6]. In Ghana, four doses of RTS,S/AS01_E_ are given at 6, 7, 9 and 24 months of age, co-administered with vitamin A at 6 and 24 months, and with measles and yellow fever vaccine at 9 months [7]. Ghana has a well-functioning EPI, with 97% national coverage for the third dose of pentavalent vaccine. However, vaccine coverage after the first year of life has proved a challenge, with coverage of Measles Containing Vaccine second dose (MCV-2), given at 18 months, currently reported at 83% [8].

This study aimed to document the challenges and lessons learned during the planning and early implementation phases of the RTS,S/AS01_E_ introduction, according to the regional and district level health service managers and frontline health workers who planned and delivered the vaccine, to assist future wider distribution of the vaccine in Ghana and elsewhere in Africa.

## Methods

A qualitative case study using pragmatist epistemology was conducted in the previous Brong Ahafo Region (now Bono, Bono East and Ahafo Regions) of Ghana, three of the six regions piloting RTS,S/AS01_E_ vaccination in Ghana. In-depth interviews (IDIs) with health professionals involved in the planning and delivery of RTS,S/AS01_E_ at the regional, district and community levels were conducted in July 2019, approximately three months after Ghana began administering the RTS,S/AS01_E_ vaccine. 

### Study sites

The study area lies in the forest transitional zone of Ghana with an estimated population of 2,660,648, whose major occupation is agriculture and related activities [9]. Malaria is endemic and perennial in the area, with a peak in transmission between April and October [10]. In 2019, the prevalence of malaria in children under five years of age in the region was estimated at 17% [11]. This burden is despite a high reported use of insecticide-treated bed nets (ITNs), with 80% of households in the region reporting ownership of at least one ITN, and 69% of children under five reported having slept under an ITN the night before the Malaria Indicator Survey in 2019 [11]. Under the national guidelines, all cases of suspected malaria are confirmed via microscopy or rapid diagnostic test, and treated using artemisinin-based combination therapy. The health facilities in the study area which provide curative and preventive services include hospitals (29), poly-clinics (6), health centres (84), clinics (113), private maternity homes (42), community-based health planning and services centres (190) and outreach points (1393) [12]. EPI services are provided as part of reproductive and child health services at all health facilities, mainly by Community Health Nurses (CHNs). Health professionals including specialist doctors, general practitioners, midwives, nurses, laboratory workers, administrative and support staff provide both curative and preventive services at the various health facilities.

### Sampling and data collection

The study area has a total of 21 sub-regional districts (10 implementing districts/11 comparator districts) participating in the MVIP. For this study, IDIs were conducted at the regional level and in two districts. Two of the 10 implementing districts were randomly selected using the random number generator function in Microsoft Excel. Within each selected district, one community health facility was selected using convenience sampling.

Purposive sampling was used to select health service managers involved in the planning and delivery of the RTS,S/AS01_E_ vaccine at the regional and district levels. Additionally, at the sub-district level, IDIs with frontline health workers involved in administering RTS,S/AS01_E_ were conducted, including with the nurses in charge of the selected health facilities and the CHNs working in the facilities. 

To conduct the IDIs, two interview guides were developed, one for IDIs with health service managers and one for IDIs with frontline health workers. The themes included in the interview guides were: decision-making processes for implementation; planning processes; current implementation of the MVIP, including monitoring and stakeholder involvement; participants’ knowledge, opinions and preferences for the malaria vaccine; measures taken to support implementation; and the challenges faced and lessons learned during the planning and implementation phases. The interview guides were piloted and revised during four IDIs with health service managers in an additional district. The interviews were conducted in English by three trained researchers. All interviews were digitally recorded and transcribed *verbatim*.

### Data management and analysis

The transcripts were imported into NVivo 12 for coding and analysis. Transcripts were anonymized but the interview number and type of stakeholder attributable to each quote were retained to assist the analysis as the stakeholder groups have varying roles and responsibilities within the planning and delivery of the vaccine, as well as different background levels of education. However, due to small numbers of participants with distinct roles, all identifiers except a number were removed in presentation of quotes to maintain anonymity. 

A framework analysis approach was used and an initial coding framework was developed based on the key themes presented in the interview guides [13]. These themes were then populated inductively with sub-themes as they were identified from the data. As an additional analysis, the challenges and lessons learned were coded onto the Consolidated Framework of Implementation Research (CFIR) [14]. The CFIR was selected as it is a commonly used, broad, theoretical framework that was developed to guide systematic assessment of factors that affect implementation across multi-level implementation contexts and which is being increasingly used in low- and middle-income countries, including implementation of vaccination programmes in Africa [15–18]. Using the CFIR as a guiding theoretical framework for the analysis allows the conceptualization of the challenges and lessons learned in a comprehensive, systematic and organized manner. The framework is valuable in providing a common language and approach to assessing the implementation of complex interventions, allowing researchers to better synthesize findings across interventions and settings and to develop an evidence base for understanding implementation. 

The CFIR has 39 constructs organized into five major domains which assess: intervention characteristics (eight constructs), inner setting (14 constructs), implementation process (eight constructs), characteristics of individuals (five constructs) and outer setting (four constructs). For the purposes of this analysis, the intervention characteristics reflect the characteristics of the RTS,S/AS01_E_ vaccine itself and its delivery within the MVIP. The inner setting encompasses the context internal to the Ghana Health Service and EPI infrastructure through which the MVIP is implemented. Individual characteristics represent the features of the individual health service managers, health workers and caregivers of recipients of the vaccine. Finally, the outer setting reflects the context external to the Ghana Health Service and EPI. 

The Standards for Reporting Qualitative Research [19] were used to ensure rigorous reporting of the study (see additional information). 

### Ethics

Ethical approval for the study was obtained from the Kintampo Health Research Centre Institutional Ethics Committee and the ethics committee of the London School of Hygiene and Tropical Medicine. Written informed consent was obtained from all the study participants.

## Results

Overall, 21 IDIs were conducted with regional and district level health service managers and frontline health workers across the study area (Table 1). The details of the challenges and lessons learned during the planning and early implementation phases of RTS,S/AS01_E_ are described below, categorized according to the CFIR domains. The challenges and lessons learned were associated with four of the CFIR domains and eight of the CFIR constructs (Table 2).

**Table 1:** Participants in the in-depth interviews (IDIs)

Type of participant	District 1	District 2	Total
Regional level health service managers	–	–	5
District level health service managers	4	4	8
Health facility nurse in charge	1	1	2
Community health nurses (CHNs)	3	3	6
Total			21

**Table 2:** CFIR domains and constructs associated with the challenges and lessons learned reported by health service managers and frontline health workers

CFIR domain	Theme	Challenges reported by participants	Lessons learned reported by participants	CFIR constructs
1.Intervention characteristics Characteristics of the RTS,S/AS01_E_ vaccine and its delivery within MVIP	Vaccine schedule	New vaccine contact in the 2nd year of life and a long gap between the 3rd and 4th doses	Deliver the 4th dose alongside MCV-2; implement additional strategies to increase 4th dose coverage	Complexity, adaptability
	Eligibility criteria	Strict age eligibility criteria that does not fully match malaria burden	Expand the age criteria	Complexity
	Pilot implementation	Challenges caused by not implementing the vaccine in all districts in the region	RTS,S/AS01_E_ vaccine should be delivered in all districts in the region	Complexity
2.Inner setting Features of Ghana Health Service and EPI infrastructure	Cold-chain resources	Inadequate cold-chain equipment at the health facility level	Improve cold-chain capacity at the health facility level	Available resources
	Transportation resources	Lack of functioning vehicles for transport at the district and health facility level	Ensure adequate funds are available for transport, including for fuel and repairs	Available resources
	Communication between implementing units	Lack of communication between implementing units at the regional and health facility levels	Hold meetings or workshops between implementing regions and between implementing health facilities	Networks and communication
	Communication and relationship between researchers and implementers	Unclear communication and understanding of the relationship between the researchers evaluating MVIP and the implementers delivering the RTS,S/AS01_E_ vaccine		Networks and communication
3.Implementation process	Sensitization	Inadequate initial sensitization	Need for a longer, more intensive and sustained delivery of contextually-appropriate sensitization prior to implementation	Planning, engaging
	Implementation timeline	Short initial timeline and delayed launch	Incorporate sufficient planning period in the timeline and ensure all logistic and financial resources are available prior to the launch date to prevent delays	Planning
	Training	Inadequate training	Hold additional training sessions following vaccine introduction; train more groups of health managers and frontline workers; include more training on practical aspects of vaccine administration	Planning, engaging
4.Characteristics of individuals Characteristics of the individuals who delivered and received the RTS,S vaccine	Vaccine uptake	Refusals to receive the RTS,S/AS01_E_ vaccine due to circulating rumours	Improve the sensitization (see above); deliver the vaccine to all districts in the region; ensure that there is no delay to the advertised launch data (see above)	Knowledge and beliefs about the intervention
	Self-efficacy of CHNs	Issues with the capabilities and confidence of CHNs to apply eligibility criteria	Hold additional training sessions and include more training on eligibility criteria (see above)	Self-efficacy
5. Outer setting Features of the context external to Ghana Health Service and EPI	Rumours	Circulating rumours about the RTS,S/AS01_E_ vaccine		Community context*

^*^We propose the addition of a new construct, community context, into the outer settings domain

### Intervention characteristics

#### Vaccine schedule

Many of the health service managers and frontline health workers described the timing of the 4th dose of RTS,S/AS01_E_, given at 24 months of age, as a major challenge. Participants explained how the 15-month gap between the 3^rd^ and 4th doses was too large for caregivers to remember to return to the vaccination clinic and that vaccinations given in the second year of life have poor coverage. Multiple participants discussed how they would have preferred this gap to be shortened, and that the 4th dose be combined with MCV-2 at 18 months.

*“The last *[RTS,S/AS01_E_ dose 4] *at 24 months, it’s too long. I was thinking at the time it could have been incorporated into the measles (MCV-2), but once it has started you can’t do anything. If implementers look at the 24 months, that time we will get a lot of drop outs.”* IDI-05

Several health service managers suggested that additional strategies would be needed to increase the coverage of the 4th dose, including mass campaigns, intensified education, targeting pre-schools, improved defaulter tracing and an award system for caregivers who bring their child for the 4th dose.

“[children receive the fourth dose]* on the condition when we intensify our education very well. And also, we go to the various places that children at that age will be, especially the creche, the day-care centres and pre-schools.”* IDI-07

#### Eligibility criteria

The strict age eligibility criteria for vaccination, which excluded children over 6 months of age from receiving the first dose of RTS,S/AS01_E_, was also discussed as a challenge. A small number of health workers reported that themselves and their colleagues would prefer that the age eligibility for the vaccine should be expanded to include all children under five years due to the burden of malaria in this age group and the difficulty in explaining to caregivers that their children just over 6 months of age are not able to receive the vaccine, when many other EPI vaccines can be given as a catch-up vaccination.

*“Mothers have been asking why? Why their children are not included, why have you neglected them? So we wish they all have this opportunity to have the vaccine.”* IDI-10

#### Pilot implementation

Challenges created by implementing RTS,S/AS01_E_ only in some districts within the region were described by many participants, including the difficulties in trying to get health professionals, and the public, to understand the reasons for the district-specific implementation. Many of the health service managers interviewed described the disappointment of the non-implementing districts, adding that there was some confusion over the selection process and that decisions may have been political, with some districts feeling that they were purposefully neglected.

“*Most of them *[non-implementing district health service managers]* are really pained, they were expecting that at the end of the day they are all going to vaccinate… For them to know that they are not part, they are really disappointed*” IDI-04

Health service managers and frontline health workers also described challenges with the vaccine coverage and eligibility criteria due to the creation of implementing and non-implementing districts within the regions. Participants reported that some children travelled from non-implementing districts to receive the vaccine, and that some children in implementing districts travelled to non-implementing areas before they could complete all four doses.

*“The difficulty has been trying to find out if the particular child coming for the vaccine actually lives in the district, so we can follow up the child receiving all 4 doses of the vaccine… some were given dosage who do not reside in the district, so some children are already going to default in receiving their second and third dosage.”* IDI-21

Participants also described how the district-specific implementation added complexity to the eligibility criteria as before administering the vaccine, CHNs have to ensure that the child resides in an implementing district, and will not travel to a non-implementing district before the end of the vaccine course or, if they do, that they will bring the child back for all doses. For these reasons, some health service managers and CHNs suggested that next time a similar programme is implemented, all districts in the region should implement it at the same time.

*“My suggestion is that next time a programme is coming, if it is a region, let’s include all the districts within so that if the person is moving from one district to another within the region the person will continue to get the services”* IDI-17

### Inner setting

#### Resources

Challenges with resources were described by the majority of health service managers interviewed. These included a lack of functioning cold-chain equipment in health facilities, including insufficient and inadequate refrigerators, vaccine carriers and thermometers. Health service managers also reported a lack of functioning vehicles and motorbikes at the district and health facility levels as a major challenge for monitoring and vaccine delivery services, with healthcare workers and other stakeholders having to spend their own money on fuel and use personal vehicles or rely on public transport.

*“It’s all about transportation and fuel… it’s a recommendation next time, when they are bringing a new vaccine, they should factor all these things, because at times you have to do by your own pocket”* IDI-14

*“The whole district that *[transportation]* is our major issue… at the directorate, all the vehicles * are broken down * and then most of the facilities too their motorbikes *[are broken down]*, so we have to rely on the public transport.”* IDI-15

#### Communication

The need for an improvement in the communication and experience-sharing between implementing areas was discussed as a lesson learned at multiple levels. At the regional level, there was a lack of communication between the other implementing regions in Ghana. Similarly, a lack of communication and experience sharing were discussed at the health facility level.

*“I suggest that workshops or meetings should be organized so that all the facilities meet and share the problems we encounter in the sub-districts, and maybe their experiences, we also get the same experiences.”* IDI-08.

The relationship and communication between the evaluating researchers and implementers within the MVIP were also discussed as a challenge at the regional level.

*“Eventually somebody writes a very big scientific paper, and we are just the small service provider fish in there, so we wonder so where do we fit in? Why do you come and talk scientific language to us? But then we will go and implement, then you will come and collect the data…people are getting very apprehensive”* IDI-06

### Implementation process

#### Sensitization

When asked about the key challenges that were faced during the planning phase, inadequate community sensitization prior to the launch of RTS,S/AS01_E_ implementation was mentioned by the majority of participants at all levels. Participants discussed how the period for sensitization was not long enough to be able to provide all the information needed to communities and for these messages to be understood.

*“What could be done is for planners to engage with communities within a lengthy period before implementation so that misconceptions and other doubts and fears associated with the vaccines that might be arising can be taken care of before the implementation”* IDI-17

A small number of health service managers mentioned that part of the reason that the initial sensitization was inadequate was because funds were insufficient and delayed. One health service manager stated that the funding issues for the social mobilization were in part due to the absence of a needs-based budget, so that what was sufficient for one district was insufficient for another.

*“Social mobilization on the Volta lake is different from social mobilization in Sunyani because you have almost 30 islands on the lake there. Now you have to go and meet them, where would you get money to buy the fuel for that boat?”* IDI-06

Other challenges reported with community sensitization included insufficient posters and brochures, and a lack of regionally appropriate content. One health service manager mentioned that if done again, a local name for the RTS,S/AS01_E_ vaccine should be created. This participant described how RTS,S, Mosquirix© and MVIP were all difficult to use and that creating a local name is important for community sensitization. Additionally, one health service manager felt that a lesson learned from the MVIP sensitization was that direct community-based sensitization was more effective than radio and mass media.

*“Mass media was something which we find out was not the golden thing that we all would think that if you go on radio everyone would hear. Many people won’t tune in. The station might be limited in coverage or just that people don’t want to listen” *IDI-06

#### Timeline

In addition to the time prior to implementation being too short for sensitization, multiple participants stated that overall, the timeline was not realistic, which forced health service managers to have to delete some of the other activities in their work programme.

Additionally, many participants discussed how the fact that the launch of the RTS,S/AS01_E_ vaccine was postponed from 1st March to 30th April due to delays in national RTS,S/AS01_E_ vaccine supply, caused challenges. Multiple participants reported on the confusion this created for both healthcare workers and caregivers as they had already been informed of the March start date. Some also described how the delay meant that the children who would have received the vaccine in March became ineligible.

*“That was challenging… because we had already sensitized mothers, made them know that we are going to give malaria vaccine in March and you know because of that some children miss out”* IDI-14

Because of this delay, one health service manager described how the eligibility criteria for the first month of MVIP had to be re-designed at the last minute so that these children could still be vaccinated, which led to further confusion surrounding the eligibility criteria. Furthermore, the delay reportedly caused problems with the competency of CHNs to carry out the vaccinations, due to a long gap between training and implementation and a high rate of staff attrition meaning that new untrained staff were moved to the health facilities in implementing areas.

*“Usually we do the training and then providers start work soon after, but because the vaccines delayed for more than a month, some providers forgot how to go about it, especially with regards to the eligibility criteria”* IDI-21

Given the challenges caused by the delay in vaccination launch, multiple health service managers suggested if done again, there should be adequate planning to ensure that the logistic and financial resources are available before the programme starts.

#### Training

Various challenges with training were commonly reported by participants. Multiple participants suggested that more regional and district health service managers should have been trained. Additionally, some frontline health workers suggested that volunteers and clinical nurses should have had formal training so that they could have better assisted the community sensitization. A few CHNs also felt that their training did not include enough content on practical aspects, such as the vaccine eligibility criteria and adverse events. Additionally, the district training sessions were only held once, and some participants mentioned that additional sessions needed to be held for CHNs who missed the training, or who joined after the initial training was held.

### Characteristics of individuals and outer setting

#### Self-efficacy of CHNs

Multiple health service managers mentioned that some CHNs did not have the capability to assess the eligibility criteria for vaccination. This lack of capability and confidence was reported to be particularly common among CHNs who joined the health facility after the training had been delivered, due to the lack of follow-up training. However, these challenges with the eligibility criteria were only reported by the health service managers interviewed and not by the CHNs themselves.

#### Rumours and refusals

The majority of the health service managers and health workers interviewed reported that some caregivers refused the vaccine due to rumours circulating about the RTS,S/AS01_E_ vaccine on social media, such as audio and video messages on WhatsApp. These rumours included statements that the vaccine had not been approved by the WHO or the Food and Drug Administration (FDA), that Europeans were using Ghanaian children as guinea pigs to test the vaccine, that the vaccine would sterilize or kill children, and that politicians and healthcare workers were taking bribes.

*“Somebody came out and said Ghanaians, don’t allow the nurses to immunize your child, we have taken bribes, somebody even fought with my in-charge that they have given us bribe… they want to kill them, we don’t want this children to give birth in the near future, so this thing in fact disturbed us a lot” *IDI-10

Despite the refusals, resistance to the vaccine during this period was described by some participants as involving just isolated cases rather than as a collective, sustained movement. Participants reported that following circulation of the rumours, more funding was released so that the community sensitization could be strengthened and intensified. Multiple participants described how the rumours and intensive sensitization that followed had not been experienced with previous vaccine introductions.

*“With the other vaccines it wasn’t so difficult as with the RTS,S because you just sensitize the public we are introducing this vaccine and they receive it so easily. But with this *[RTS,S],* we really had to do sensitizing I tell you.” *IDI-01

Participants discussed multiple different factors that they believed contributed to the rumours spreading and leading to people refusing the vaccine.

*“Planners didn’t do a lot of sensitization before implementing the vaccine. The timeframe between the actual sensitization and implementation was very short. So, that did not allow people to learn more about the vaccine before the implementation, I think that is one of the reasons why there are misconceptions about the vaccine.” *IDI-17

Some implementers also mentioned that the design of the RTS,S/AS01_E_ implementation as a pilot study gave weight to the rumours. Participants suggested that the use of the word ‘pilot’, along with the fact that not all districts and regions were implementing, and that verbal autopsies were being carried out as part of the evaluation seemed to suggest that rumours that the malaria vaccine was an experimental one were correct.

*“districts that are left out in the program should be called in immediately, because, there are messages in other villages that is circulating in social media that are portraying that this vaccine we are using it to sterilize children, it is not safe, people shouldn’t go for it, and that is why the whole country is not into it and some few districts are selected” *IDI-17

The delayed launch also led to general feelings of distrust, with some community members questioning whether they were being deceived because the vaccine had not come when they were told it would. Additionally, one health service manager added that the Ebola vaccine trial suspension following rumours and national controversy in Ghana in 2015 contributed to the rumours spreading.

#### Relationship between challenges and lessons learned

**Fig. 1:**
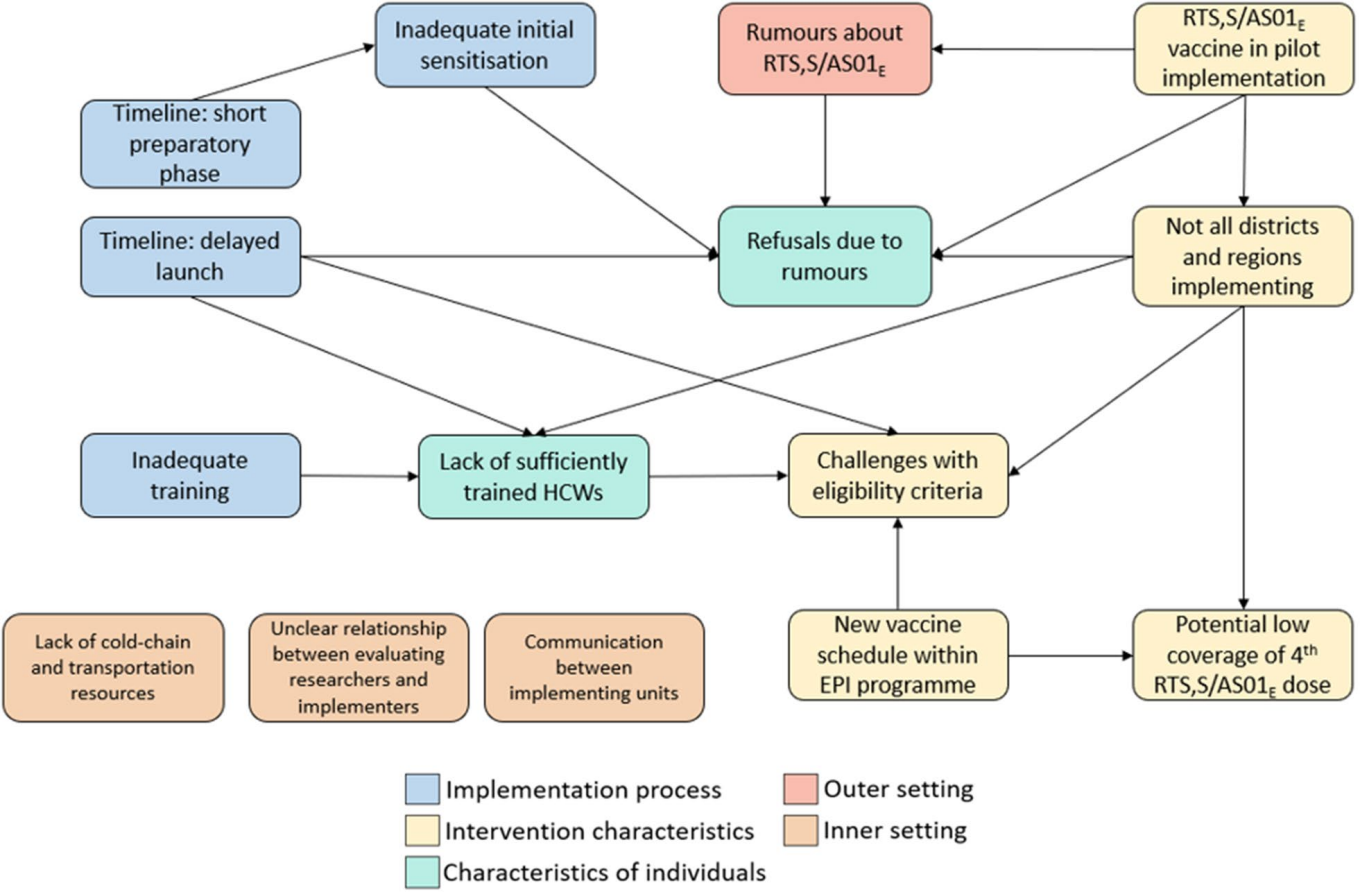
The relationship between the challenges and lessons learned identified, categorized by CFIR domain

While this paper presents the challenges and lessons learned within the separate domains of the CFIR, our data clearly showed the relationships between challenges within and between different domains (Fig. 1). The challenges experienced were highly inter-connected; in particular, the challenges associated with the implementation process and with the intervention being a pilot programme were a contributing factor to many of the other challenges. No connections between the challenges associated with the inner settings were apparent.

## Discussion

This study identified several key challenges and lessons learned by health service managers and frontline health workers during the planning and early implementation phases of the RTS,S/AS01_E_ vaccine. These included challenges and lessons learned associated with the nature of the intervention itself, how it was implemented, and the systems and contexts within which it was implemented.

A major challenge reported by the majority of participants was the impact of rumours that originated on social media during the first month of implementation; the health service managers and health workers had not faced challenges with rumours for any previous EPI vaccines and this challenge, along with the intensive community sensitization needed to counter the rumours, was seen as unique to the RTS,S/AS01_E_ vaccine introduction. Some participants suggested that factors external to the programme may have contributed to the impact of the rumours, such as the rumours and controversy surrounding the Ebola vaccine trial suspension in Ghana in 2015 [20]. Other factors seen to significantly contribute to the impact of the rumours included the two-month delay in the launch of the vaccine and the initial inadequate sensitization. This study highlights the need for a longer, more intensive and sustained sensitization period prior to implementation in similar vaccine introductions, as well as continuing intensive education and mobilization during the implementation phase. This sensitization should include locally-appropriate content and be delivered primarily through community-based communication channels. Additionally, to counter the circulation of rumours on social media, community sensitization messages could also be delivered through social media platforms, including WhatsApp, as has been suggested for the COVID-19 vaccination campaign in Ghana [21, 22].

Previous studies of vaccine introduction in LMICs have commonly reported the challenges resulting from limited social mobilization activities, noting a strong social mobilization delivered within a realistic timeframe as a key lesson learned [23–25]. Additionally, distrust caused by delays in vaccine introductions have also been documented previously [25].

Features associated with the RTS,S/AS01_E_ implementation being a pilot programme were found to have affected the impact of rumours. The fact that implementation was only happening in some districts and regions and even the use of the word ‘pilot’ seemed to lend credence to the rumours that the vaccine was unsafe, not approved by the WHO or the FDA, and was being trialled in Ghanaian children. Additionally, the verbal autopsies conducted as part of the vaccine evaluation were also seen to support these rumours, despite the fact that they have been carried out for many years in the study area. The pilot study design also added further complexity to the eligibility criteria, as it meant CHNs could only give the RTS,S/AS01_E_ vaccine to children who lived in the implementing areas, and had to try to ensure that these children would remain in the implementing areas to receive the remaining three doses. Participants reported that families in the study area commonly travel to neighbouring districts, including non-implementing areas for seasonal work. The age eligibility criteria was also reported as being more complicated than for other EPI vaccines due to the fact that despite the malaria burden in the age group, children over 6 months of age are not able to receive the first dose of the vaccine, while most EPI vaccines can be given as a catch-up.

The implementation design contributed to the lowered capacity of CHNs at the health facilities to apply the eligibility criteria, as CHNs from non-implementing areas who had not received any training on MVIP were moved into implementing health facilities to replace CHNs who had left, compounded by the delay in launch date. The high rate of attrition of healthcare workers is a documented weakness of the Ghanaian EPI, and therefore should be considered when planning for timely implementation [26]. Early national level MVIP monitoring reports showed that issues with coverage were partly due to knowledge gaps on the eligibility criteria, particularly among newly recruited frontline staff, showing that this issue extends beyond the study area [27]. These are important lessons for the introduction of other interventions through pilot studies.

There is, however, evidence to suggest that some of the initial challenges experienced have been overcome. Twenty-four months after the launch of MVIP, uptake of RTS,S/AS01_E_ is high with around 70% coverage for RTS,S/AS01_E_ doses 1 and 3, with preliminary qualitative data showing caregivers’ concerns related to rumours and the district/region-specific implementation have markedly declined compared to earlier [28].

Both the health service managers and health workers voiced strong concern over the timing of the 4th dose at 24 months, 15 months after children receive the 3rd dose. Since the 1970s, EPI programmes in Africa have focussed on children below 12 months of age. MCV-2 was recently introduced as one of the first childhood vaccines delivered beyond this age, with significantly lower coverages than for MCV-1 [29]. Participants proposed intensified sensitization and education, defaulter tracing, targeting of day-care centres and setting up an awards system as potential strategies to increase the coverage of the 4th dose. These suggestions are supported by a study addressing the challenges of immunizing in the second year of life in three regions in Ghana [30]. An additional strategy suggested by the participants to increase coverage is to deliver the 4th dose using mass vaccination campaigns. Given the promising results from a recent trial investigating the use of seasonal RTS,S/AS01_E_ vaccination in young children in areas with highly seasonal malaria transmission, mass campaigns for the 4th dose could be timed to the peak malaria transmission season to increase the effectiveness of the vaccine [31].

While some challenges for the pilot programme were seen as being specific to MVIP, other key challenges reported were similar to those experienced in previous EPI vaccine introductions in Ghana, including challenges related to weaknesses in the overall EPI infrastructure [26]. Transportation constraints, especially at the district and health facility levels, and inadequate capacity and regular break-down of cold-chain equipment at the lower levels of the health system, are documented weaknesses of the Ghanaian EPI system, and have also been documented for other LMIC vaccine introductions [26, 29, 32]. Additionally, other studies have reported that insufficient available funds for transport at the district level in Ghana, including for vehicle maintenance and fuel, impact the ability of district health managers to carry out their planned activities [33, 34]. Furthermore, issues with training are often a documented challenge in new vaccine introduction programmes in LMICs, and the post-introduction evaluation for MCV-2 in Ghana also reported that healthcare workers were inadequately trained regarding eligibility criteria [26, 35].

This study identified 12 main challenges and lessons learned that were associated with the five CFIR domains and nine of the constructs. While most of the challenges fitted well within the original framework, there was some uncertainty where the challenges due to rumours should fit. While this challenge partially fits within the domain ‘characteristics of individuals’, it went beyond the individual level and could also fit within the outer settings domain, but was not associated with any of the four constructs in this domain. A previous study by Escoffery et al. [36] also placed misinformation into the outer setting. For this reason, the outer settings domain of the CFIR were modified by adding the construct ‘community context’ to accommodate rumours and misinformation. Rumours and misinformation that circulate in communities pose significant threats to the effective implementation of health interventions, particularly given current widespread access to and use of social media [37]. Therefore, it is important that this is captured within the CFIR, as well as other contextual factors in communities that effect implementation. Means et al. [18] made a similar suggestion, proposing the addition of a construct called community characteristics into the outer setting.

One strength of using this framework to categorize the challenges and lessons learned is that it allows reflection on which of these challenges would likely be replicated in other implementation programmes and which parts of the intervention can be adapted to attempt to avoid these challenges. For example, while the challenges associated with the implementation process, characteristics of individuals and inner and outer settings may be more specific to what occurred during the RTS,S/AS01_E_ implementation in Ghana, those associated with the characteristics of the intervention would likely occur in other similar implementation settings. Furthermore, as there is less potential for variation in these core parts of the intervention, it is the supportive interventions, such as the training and sensitization, that can most easily be adapted to mitigate the challenges identified in this study.

While this study identified many practical lessons from the planning and implementation of the RTS,S/AS01_E_ vaccine, the generalizability of the results should be interpreted with caution. In this study, health facilities were selected using convenience sampling and the districts were randomly selected, which likely missed some variation in health facility and district experience of RTS,S/AS01_E_ implementation and it is possible that some challenges unique to certain areas were missed. Additionally, the findings of this study may not reflect those of the other two implementing regions within Ghana. However, it is likely that there are many similarities as the MVIP is a nationally coordinated programme. Similarly, not all of the findings from this study will be generalizable to the other two implementing countries. A further limitation is that interviews were coded by a single researcher. The analysis was therefore inevitably shaped by the lens through which this researcher interpreted the data. However, the analysis was reviewed by the other researchers who conducted the interviews, as well as the researchers at KHRC who were very familiar with the implementation of the RTS,S/AS01_E_ vaccine in the region, which helped to ensure the credibility and confirmability of the findings.

## Conclusion

Health service managers and frontline health workers in three of the six regions included in the MVIP in Ghana faced numerous challenges during the planning and early implementation of the RTS,S/AS01_E_ vaccine. These challenges were related to the nature of the RTS,S/AS01_E_ vaccination schedule, the choice of implementing the vaccination as a pilot programme, the process of implementation, and the features of the systems and contexts within which it was implemented. These challenges were found to be highly connected, with issues in one domain contributing to other challenges. Key lessons learned from the implementation of RTS,S/AS01_E_ include the need for a more intensive and sustained period of community sensitization prior to implementation and for adequate planning to occur to ensure no delay in between stated and actual implementation.

After reviewing the evidence from MVIP, on 6th October 2021, WHO formally recommended the use of the RTS,S/AS01_E_ vaccine for children living in regions with moderate to high malaria transmission [6]. Therefore, the insights on the challenges and lessons learned during the MVIP described in this study should be helpful to immunization programme managers when considering the likely scale-up of RTS,S/AS01_E_ within Ghana and possible implementation in other sub-Saharan African countries. These findings are also of potential relevance to other future vaccine introductions, in particular the challenges describing the issues related to pilot introduction programmes.

## Data Availability

The datasets generated and analysed during this study are not fully available as it is not possible to fully anonymize the transcript files. The transcript files do not include the names or job titles of participants, however, some of the files would be identifiable to a person familiar with the context given that study participants speak in detail about their experiences implementing the malaria vaccine in the study area, and their responses are intrinsically shaped by their specific roles and responsibilities within the implementation.

## Abbreviations

CFIR: Consolidated Framework for Implementation Research
CHN: Community Health Nurse
EPI: Expanded Programme of Immunizations
FDA: Food and Drug Administration
IDI: In-depth interview
ITN: Insecticide-treated bed net
LMIC: Low and Middle Income Countries
MCV-2: Measles Containing Vaccine dose 2
MVIP: Malaria Vaccine Implementation Programme
WHO: World Health Organization
